# Self-calibration strategies for reducing systematic slope measurement errors of autocollimators in deflectometric profilometry

**DOI:** 10.1107/S1600577524003552

**Published:** 2024-06-05

**Authors:** Ralf D. Geckeler, Andreas Just, Michael Krause, Olaf Schnabel, Ian Lacey, Damon English, Valeriy V. Yashchuk

**Affiliations:** a Physikalisch-Technische Bundesanstalt Bundesallee 100 38116Braunschweig Germany; b Schnabel Elektronische Messtechnik, Rosengarten 10, 22880Wedel, Germany; c Advanced Light Source Lawrence Berkeley National Laboratory Berkeley CA94720 USA; d Engineering Division Lawrence Berkeley National Laboratory Berkeley CA94720 USA; European XFEL, Germany

**Keywords:** angle metrology, autocollimator, optical metrology, form measurement, deflectometry, profilometry, data processing, error suppression, synchrotron, free-electron laser

## Abstract

Deflectometric profilometers are used to precisely measure the form of beam shaping optics of synchrotrons and X-ray free-electron lasers. They often utilize autocollimators which measure slope by evaluating the displacement of a reticle image on a detector. Based on our privileged access to the raw image data of an autocollimator, novel strategies to reduce the systematic measurement errors by using a set of overlapping images of the reticle obtained at different positions on the detector are discussed. It is demonstrated that imaging properties such as, for example, geometrical distortions and vignetting, can be extracted from this redundant set of images without recourse to external calibration facilities. This approach is based on the fact that the properties of the reticle itself do not change – all changes in the reticle image are due to the imaging process. Firstly, by combining interpolation and correlation, it is possible to determine the shift of a reticle image relative to a reference image with minimal error propagation. Secondly, the intensity of the reticle image is analysed as a function of its position on the CCD and a vignetting correction is calculated. Thirdly, the size of the reticle image is analysed as a function of its position and an imaging distortion correction is derived. It is demonstrated that, for different measurement ranges and aperture diameters of the autocollimator, reductions in the systematic errors of up to a factor of four to five can be achieved without recourse to external measurements.

## Abstract 2

The beam shaping optics of synchrotrons and X-ray free-electron lasers are routinely measured using deflectometric profilometers, which use autocollimators to evaluate the surface slope from the displacement of a reticle image on a detector. Here, novel strategies to reduce systematic measurement errors by using a set of overlapping images of the reticle obtained at different positions on the detector, which can reduce the systematic errors by up to a factor of four to five without recourse to external measurements, are discussed.

## 1. Introduction

Commercial electronic autocollimators are widely used in deflectometric profilometers for precision shape measurements of optical surfaces, especially X-ray mirrors. The concept of an autocollimator-based optical deflectometer was first proposed and realized at the Physikalisch-Technische Bundesanstalt (PTB) by E. Debler and K. Zander in 1978–1980 and by K. von Bieren in 1985 (Debler & Zander, 1980 ▸; Von Bieren, 1985 ▸). Currently, a growing number of laboratories at synchrotron and free-electron laser X-ray facilities are using autocollimator-based profilometers which are similar in design to the BESSY-II NOM (Siewert *et al.*, 2004 ▸, 2010 ▸, 2014 ▸). Most, if not all, of these profilometers are based on various applications of the Elcomat series of autocollimators manufactured by Moeller Wedel Optical, Germany. These devices have proven capable of characterizing state-of-the-art aspheric X-ray optics to an accuracy in slope of the order of 50 nrad (root-mean-square, RMS); see, for example, Alcock *et al.* (2010 ▸), Yashchuk *et al.* (2010 ▸), Lacey *et al.* (2014 ▸, 2018 ▸), Assoufid *et al.* (2013 ▸), Nicolas & Martínez (2013 ▸), Qian *et al.* (2015 ▸) and Qian & Idi (2016 ▸), and references therein.

When a surface under test (SUT) reflects the measuring beam of the autocollimator back into its objective, an image of the reticle is created in the focal plane of the objective. The autocollimator’s objective acts as an optical lever and converts the angular deflection of the beam into an image shift which is proportional to the focal length of the objective and to the tangent of the angle. The angle measurement of an autocollimator is therefore based on evaluating the shift of an image on a detector and, in the case of commercial CCD detectors, a level of accuracy of at least two to three orders of magnitude smaller than the typical pixel size is required.

Our research has shown that the performance of the algorithms used to perform this task is critical and determines the repeatability and systematic errors of the angle metrology achievable with autocollimators (Schumann *et al.*, 2016 ▸). This is especially the case for, but not limited to, angle measurement errors on an angular scale which corresponds to the pixel size of the CCD and to the size of features of the reticle pattern. The reticle pattern, however, also offers opportunities for reducing these errors (Schumann & Geckeler, 2007 ▸; Fütterer, 2005 ▸, 2007*a* ▸,*b* ▸). In this paper we consider the design of the reticle as a given, since we are dealing with a commercial autocollimator, and focus on optimizing the algorithm that determines the position of the reticle image on a detector which consists of discrete pixels.

In addition to the algorithms which evaluate the image shift, angle metrology with autocollimators is affected by the alignment of the internal optical components of the autocollimator and their imperfections. The resulting systematic measurement errors of autocollimators are highly dependent on the measurement conditions, such as the reflectivity and curvature of the SUT (Geckeler *et al.*, 2010 ▸); the autocollimator’s beam length which varies substantially when the SUT is scanned by using a movable pentaprism (Yashchuk *et al.*, 2007 ▸, 2016 ▸; Geckeler & Just, 2008 ▸); the shape, diameter and position of the aperture stop (Qian *et al.*, 2015 ▸; Geckeler *et al.*, 2010 ▸, 2016 ▸; Geckeler & Just, 2007 ▸; Lacey *et al.*, 2019 ▸; Grubert *et al.*, 2019 ▸); and the sagittal beam deflection perpendicular to the main measuring direction which results in crosstalk (Geckeler *et al.*, 2012 ▸; Kranz *et al.*, 2015 ▸; Schumann *et al.*, 2019 ▸). Environmental influences, predominantly of the air pressure and temperature, also need to be taken into account (Geckeler *et al.*, 2018*a* ▸,*b* ▸, 2019 ▸).

Systematic measurement errors caused by the alignment of the opto-mechanical components of the deflectometric profilometer setup are also an issue. In order to minimize these, we have developed procedures that allow the *in situ* angular adjustment of the components (autocollimator, SUT, pentaprism) with respect to each other, including the optical surfaces of the reflective pentaprism (Geckeler, 2007 ▸; Barber *et al.*, 2011*a* ▸,*b* ▸). Further improvements are possible by implementing sophisticated data acquisition and processing techniques that allow to anticorrelate the systematic errors of multiple repeatable measurements made in different arrangements of the measurement setup, thus suppressing the errors in the averaged trace; see, for example, McKinney *et al.* (1993 ▸), Irick (1998 ▸), Yashchuk (2009 ▸), Polack *et al.* (2010 ▸) and Yashchuk *et al.* (2013 ▸, 2018 ▸), and references therein.

In this paper, based on our access to the image data of an autocollimator, we discuss novel strategies to significantly reduce the systematic measurement errors of this device. In Section 2, we outline our reticle image processing algorithm which is based on a cross-correlation analysis of a recorded reticle image with a reference image to precisely determine the position of the recorded image relative to the reference image. In order to increase the sensitivity to the relative position shift, before the cross-correlation function is calculated, both images are resampled by interpolating the pixel­ated images taken with the autocollimator detector to a more densely sampled grid with an increased number of subpixels. As we demonstrate, the inclusion of this crucial step results in a significant increase in accuracy. In Section 2, we will also address the issue of selecting the optimal number of subpixels by use of synthetic and experimental reticle image data.

In Section 3, we show that imaging properties, specifically geometrical distortions and vignetting, can be extracted and corrected by use of a suitable set of reticle images without recourse to external calibration data. This approach is based on the fact that all observed changes in the reticle image at different positions on the detector are due to the imaging process itself. Changes in the intensity of the reticle image as a function of its position on the CCD enable us to derive a vignetting correction while changes in the size of the image provide an imaging distortion correction.

In Section 4, we apply our algorithms to experimental imaging data obtained with an autocollimator Elcomat 3000 by Moeller Wedel Optical (https://www.haag-streit.com/moellerwedel-optical/products/electronic-autocollimators/elcomat-series/elcomat-3000/). Extensive data sets covering angular measurement ranges of ±1500 arcsec, ±30 arcsec and ±4 arcsec, and aperture sizes of 32 mm, 5 mm, 2.5 mm and 1.5 mm, are analysed. Reference angle measurements obtained with PTB’s primary angle standard WMT 220 (Probst *et al.*, 1998 ▸) are used to evaluate the improvement achieved by use of our algorithms and correction methods. For a circular aperture of 2.5 mm diameter, the most commonly used in autocollimator-based surface slope profilometry, the improvement in systematic errors reaches a factor of four to five. We believe that the results of our investigations are helpful for reaching fundamental metrological limits in deflectometric profilometry using state-of-the-art electronic autocollimators.

## 2. Correlation and interpolation-based reticle image localization (CIRIL)

### 2.1. Description of the algorithm

As noted in the *Introduction*[Sec sec1], the angle measurement of an autocollimator is based on evaluating the shift of an image on a detector and, therefore, the performance of the algorithms used for this task has a critical impact on the angle metrology achievable with these devices. In this section we describe an original algorithm that allows the position of the reticle image on a detector consisting of discrete pixels to be accurately determined. We would like to emphasize that the developed algorithm can be applied to pixelated images of patterns in general, and that the experimental verification of the technique using the reticle image of an autocollimator is only a specific use case.

The central idea is to use a cross-correlation (covariance) analysis between the reticle image under test and a reference image (recorded, for example, at the centre of the measurement range) for the precise determination of the position of the image on the detector. This concept is well established, but we have added a crucial step. Before calculating the cross-correlation, both images are resampled by interpolating the pixelized images to a denser grid of sampling points by dividing each pixel into a number of *N* subpixels. As we will demonstrate, it is this combination of interpolation and cross-correlation that enables us to determine the relative shift of two reticle images relative to each other with increased accuracy which depends on the noise level of the image and thus on the aperture of the autocollimator.

We assume a reticle image given by discrete intensity values for each detector pixel, *I*_*k*_, *k* ∈ [1,…, *K*]. We also assume that a reference image is selected, with 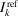, *k* ∈ [1,…, *K*]. Each detector pixel is then divided into *N* subpixels and a linear interpolation of each reticle image to this expanded sampling grid is performed, resulting in intensity values 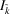, 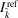, 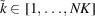. We now shift the interpolated intensity pattern of the reticle image under test, 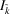, by a number of 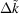 subpixels and calculate the covariance of it and the interpolated intensity pattern of the reference image, 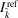. For an easier mathematical notation, we assume a cyclical shift of the interpolated image under test and its associated indices, which results in a cross-correlation function 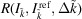 that is proportional to the covariance 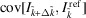, specifically
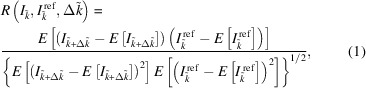
where 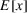 denotes the expectation value of the values in parentheses and cov[*x*,*y*] denotes the covariance, which is defined as
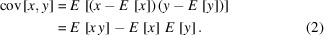
In the general (non-cyclical) case, the calculation of the covariance has to be restricted to the overlapping indices of the two intensity patterns.

Now that the cross-correlation function 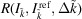 is available, a suitable method for determining its maximum with respect to 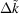 has to be implemented. To this purpose, a local polynomial fit to the cross-correlation is performed within a moving window of half-width 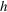 subpixels. To be more specific, for shifts 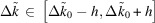, a polynomial 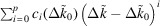 is fitted to the cross-correlation values 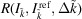. The coefficients 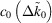 are used to find an approximation for the position of the maximum of the cross-correlation function. The coefficients 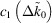 are then interpolated linearly to determine the position with 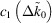 = 0 more accurately. The two-step procedure allows to minimize the known bias error of the position evaluation; see, for example, Yashchuk (2006 ▸).

Finally, multiple tests of the parameters of the algorithm (number of subpixels *N*, half-width *h* of the window for the local polynomial fit in units of subpixels, and order *p* of the polynomial) were performed to validate the educated guesses on which their selection was initially based. In the following Section 2.2 we demonstrate that *N* = 10 is adequate. For this number of subpixels, *h* = 4 and *p* = 3 are the preferred choices. This choice of parameter resulted from a comparison of the performance of the CIRIL algorithm with the reference measurements provided by the WMT 220 primary angle standard. However, it was found that the influence of the choice of the parameters on the results was small compared with the overall improvement provided by the application of the CIRIL algorithm. For different aperture diameters of the autocollimator, variations in the parameters *h* and *p* resulted in changes in the uncertainty of the image shift between 1% and 10%. In contrast, the choice of *N* = 10 subpixels instead of *N* = 1 (*i.e.* no interpolation of the images before cross-correlation) resulted in an improvement by approximately two orders of magnitude, which we demonstrate in the following section.

### 2.2. Testing of optimal number of subpixels

In Section 2.1, we describe in detail how our algorithm divides each pixel into a number of *N* subpixels and interpolates the imaging data before performing a cross-correlation analysis between the image under test and a reference image. In this section, we demonstrate that, by combining interpolation and correlation in this way, the relative shift between the image under test and the reference image can be evaluated far more accurately compared with a correlation analysis without a preceding interpolation of the images to a denser sampling grid.

We begin the investigations on the capabilities and fundamental limits of the proposed algorithm with the help of synthetic reticle images. For this purpose, a ten-slit reticle was simulated with the design parameters of the Elcomat 3000 series autocollimator. This is the autocollimator used to obtain the experimental data, the analysis of which is presented in Sections 4 and 5 in detail. (Note that the exact design specifications of the reticle are subject to non-disclosure by the manufacturer; therefore, we are not able to provide detailed information on them in this paper.) We shifted the synthetic images with respect to the CCD pixels virtually by fractions of a pixel and integrated the intensity within each detector pixel. This is an idealization. The pixels of the CCD detector feature intra-pixel variations in their quantum efficiency due to their internal electrode structure. For evaluating the fundamental limits of the algorithm under idealized measuring conditions, we have also neglected the influence of optical aberrations.

The three-cornered hat (3CH) method (Gray & Allan, 1974 ▸) can be used to characterize measurement data sets that contain identical systematic measurement errors that are common to all data sets and are not relevant to the characterization. It is commonly used to analyse the time signals of clocks and allows the evaluation of their respective frequency stabilities. The advantage of the 3CH method is that a systematic measurement deviation which is common to all data sets is eliminated, since the method is based on the analysis of the differences between all pairs that can be formed from each subset of three data sets.

We assume three sets of measurement data, *y*_*ai*_, *y*_*bi*_ and *y*_*ci*_, *i* ∈ [1,…, *M*], as follows
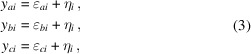
whereby the η_*i*_ are systematic measurement deviations which are common to all data sets, and ɛ_*ai*_, ɛ_*bi*_ and ɛ_*ci*_ are (random) measurement errors associated with each measurement. The differences between two sets of measurement errors, for example ɛ_*ai*_ and ɛ_*bi*_, then feature the following statistical property,
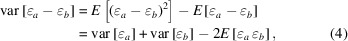
where *E*[*x*] denotes the expectation value of the values in parentheses and 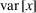 denotes their variance. We assume that the measurement errors are all uncorrelated, *i.e.*
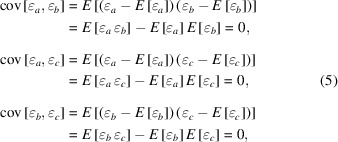
where 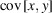 denotes the covariance of the values in parentheses. By using this assumption and by assuming *E*[ɛ_*a*_] = *E*[ɛ_*b*_] = *E*[ɛ_*c*_] = 0, we can simplify equation (4) further, as follows,
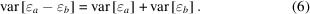
By calculating differences between all pairs which can be formed from the three data sets, we can then derive the variances of the differences,
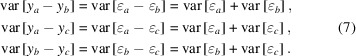
The solution to the equation system is then given by
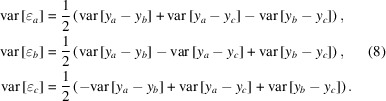
An alternative analysis of the statistical properties of the data sets can be based on the Groslambert covariance (Groslambert *et al.*, 1981 ▸; Fest *et al.*, 1983 ▸) by using the following relations,
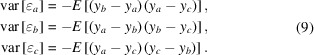
The relations (9) can be derived from the equations given above, including the assumptions that the errors ɛ are uncorrelated and that their expectation values are zero, such as is commonly the case with random measurement errors. While this might not be strictly the case in our applied case, the 3CH method should nevertheless provide a usable *in situ* estimate for the variance of the error associated with each data set without recourse to external comparison data as it obviates the need to subtract the systematic errors that are common to all data sets (*i.e.* the systematic measurement deviations of the autocollimator). Note that the final analysis of the experimental data does include a comparison with data provided by an external reference system. It is just that the evaluation of the error propagation properties of the algorithms is facilitated by applying the 3CH method for first tests.

**Figure 1:**
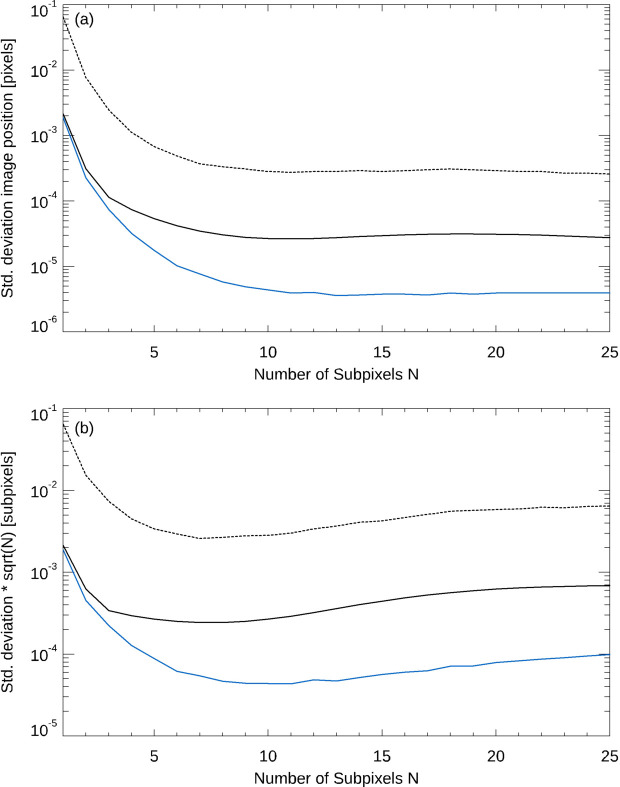
Standard deviation of the position of the reticle image under test relative to a reference image, evaluated by our CIRIL algorithm, as a function of the number of subpixels *N*. The standard deviation is presented in units of pixels (*a*) and subpixels (*b*), see text for details. Solid blue line: synthetic reticle images. Solid and dashed black lines: experimental data obtained by using an autocollimator aperture of 32 mm (moderate level of imaging noise) and 2.5 mm (high noise level), respectively.

First, we make use of the synthetic image data as described above. Fig. 1 ▸(*a*) shows the standard deviation (specifically, the square root of the variance evaluated by the 3CH method) of the position of the synthetic reticle image under test relative to a reference image, evaluated by our CIRIL algorithm, as a function of the number subpixels *N* (solid blue line). To demonstrate that a similar improvement is also achieved in the case of experimental data, the solid and dashed black lines show the standard deviations associated with the application of the same algorithm to experimental data. We have chosen a data set which was obtained by using the full aperture of the autocollimator (Elcomat 3000 SN 975, *X*-axis, aperture 32 mm, SUT distance 300 mm, measurement range ±1500 arcsec), because, in this case, the reticle images are the closest to the synthetic images (solid black line). The use of the full aperture corresponds to an illuminated aperture of the outgoing measuring beam of approximately 32 mm in diameter, while the diameter of the autocollimator’s objective is 50 mm.

With both synthetic and experimental data, a decrease in the standard deviation of the position as a function of *N* is observed. In the case of the experimental data, however, imaging noise limits the achievable standard deviation of the position evaluation and leads to a base value for it that is not undercut when *N* is increased. In order to support this interpretation, we have added the standard deviation associated with the application of the CIRIL algorithm to a second experimental data set to Fig. 1 ▸(*a*) (dashed black line). It was obtained by using a circular aperture with an autocollimator aperture of 2.5 mm in diameter, as compared with 32 mm for the first experimental data set. This results in an even more pronounced influence of the imaging noise and a more elevated base value for the standard deviation for large number of subpixels *N*. Fig. 1 ▸(*b*) shows the standard deviations σ(*N*) of the reticle image positions from (*a*) after they have been rescaled according to σ(*N*) · *N* so that they are expressed in units of the dimensions of the subpixels, rather than in units of the pixels. This graph demonstrates that the reduction in standard deviation is not achieved by the interpolation of the reticle image to a denser sampling grid as such, but by the interaction of the cross-correlation with the image interpolation. As the standard deviation associated with the experimental data does not improve substantially for *N* ≥ 10 and since the computing time increases with *N*, *N* = 10 was used for further analyses of the experimental data in this paper.

## 3. Reticle imaging self-calibration (RISC)

### 3.1. General approach

In this section, we are guided by the following question: can a set of reticle images be used to extract properties of the imaging (such as, for example, geometrical distortions and vignetting caused by the autocollimator’s objective, quantum efficiency variations of individual CCD pixels, geometry deviations of the CCD, *etc*.) without recourse to external measurements? This idea is based on the fact that the properties of the reticle itself do not change – all changes in the reticle image are due to the imaging process. For a large calibration range, the reticle images overlap and provide us with ample redundant data.

For our proof-of-principle tests, we have applied the local polynomial fit from Section 2.1 to the image data which provides the position of each individual slit of the autocollimator’s reticle and the intensity at the position. We are well aware that advanced strategies might be utilized which allow an even more optimal error separation based on redundancy, with the shearing method being a prominent example that we have applied to various metrology problems extensively in the past (Elster & Weingärtner, 1999 ▸; Elster, 1999 ▸, 2000 ▸; Geckeler *et al.*, 2006 ▸, 2014 ▸; Geckeler & Just, 2014 ▸).

Similarly to the procedure applied in Section 2.1 to determine the position of the maximum of the cross-correlation function, the local polynomial fit to the intensity values of the image is performed within a moving window of half-width *h*′ pixels. For image pixels *k* ∈ [*k*_0_ − *h*′, *k*_0_ + *h*′], a polynomial 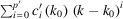 is fitted to the intensity values of the image. The coefficients *c*_0_(*k*_0_) are used to find an approximation for the position of the local maxima of the intensity values of each slit of the reticle and the coefficients *c*_1_(*k*_0_) are then interpolated linearly to determine the positions with *c*_1_(*k*_0_) = 0 more accurately. The corresponding intensity values are obtained by interpolating the intensity values of the image at the positions of the local maxima. The parameters *h*′ = 6 and *p*′ = 3 were used for this local fit. Similarly to the consideration in Section 2.1, the influence of the parameters on the results of the reticle intensity analysis are minimal.

### 3.2. Flat-field correction

In the case of optical imaging, various influences alter the intensity of the image, such as vignetting by the objective, which is characterized by a decrease in image brightness towards the periphery of the image plane compared with the centre. In addition, dust can be deposited on the CCD detector or its protective glass cover, and the detector itself usually exhibits consistent differences in quantum efficiency between pixels (*i.e.* individual responses of pixels to the same amount of light). In astronomical imaging, correction for these effects is achieved by a process called flat-fielding, which relies on imaging an object with uniform illumination, such as an illuminated screen mounted inside the dome at which the telescope is pointed.

The aim of our approach is to use the varying intensity of the reticle image as it moves across the image field to derive a flat-field/vignetting correction. Specifically, for each individual reticle slit, the intensity value at the position of the slit on the CCD was derived as a function of position. Section 3.1 describes in detail how the position and intensity of each slit is determined. The overlap between the data sets is used to correct for global differences in the intensity values for each slit by scaling them accordingly (*i.e.* the intensity values for each slit were multiplied by a constant correction factor). The intensities were then averaged by binning (bin size 5 pixels) and a flat-field correction was calculated for each CCD pixel by interpolation. This approach was chosen because it is capable of averaging an arbitrary number of repeated angle scans with slightly different sampling points.

In Section 4 we limit ourselves to demonstrating the effect of the correction on the angle measurement error of an autocollimator. Therefore, this section presents the results for the data set with the largest flat-field correction as an example. For this purpose, we have chosen the data set (Elcomat 3000 SN 975, *X*-axis, aperture 1.5 mm, SUT distance 300 mm, measurement range ±1500 arcsec) obtained by using the smallest aperture in combination with the largest measurement range, as this maximizes the magnitude of the vignetting. It should be noted that the autocollimator measurement range specified by the manufacturer is ±1000 arcsec and that we have extended the range beyond this to maximize the effects to be studied. The data in this paper are therefore not representative of the performance of the instrument under the measurement conditions specified by the manufacturer.

**Figure 2:**
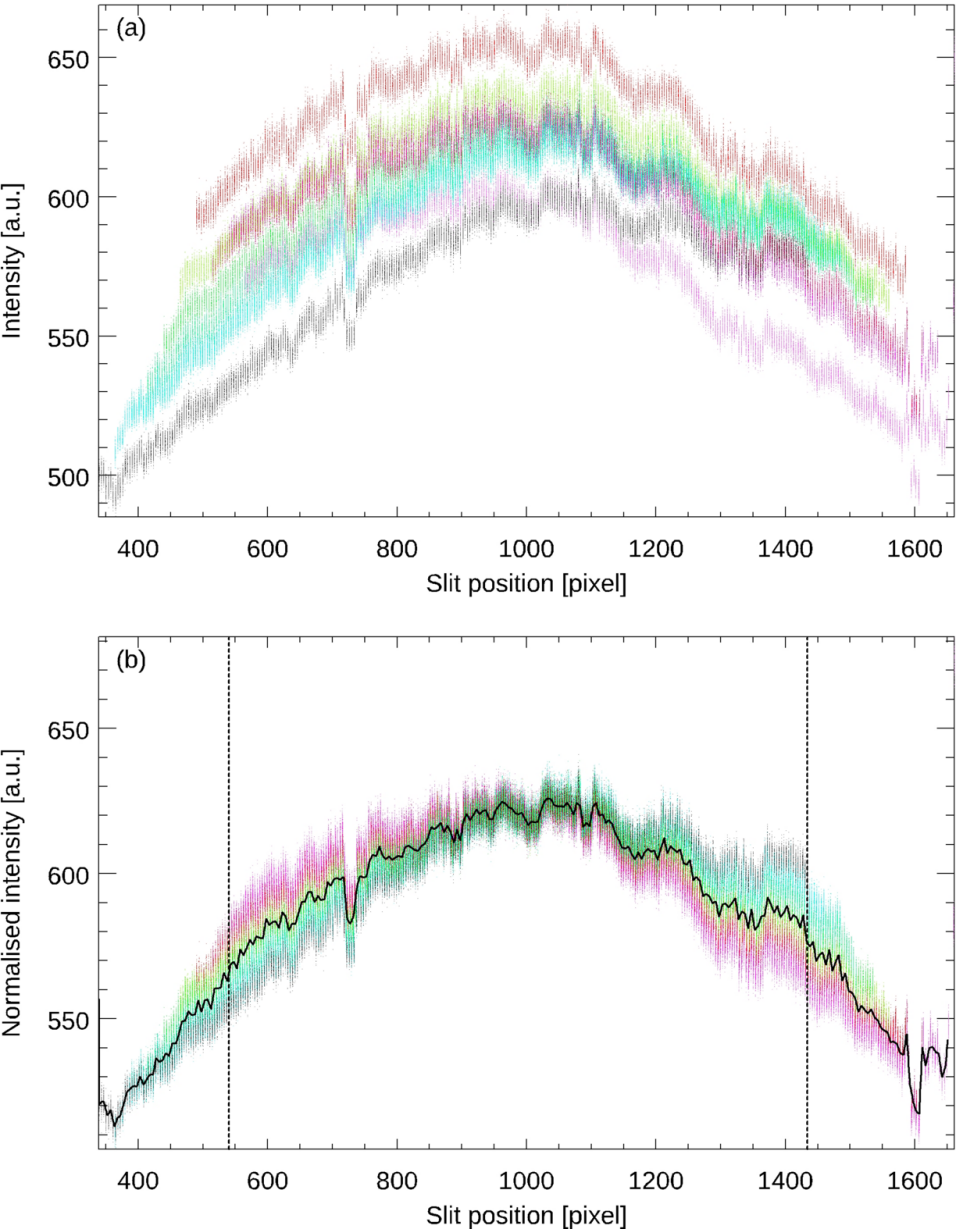
Intensity of the reticle image of an autocollimator at the position of each individual slit (colour coded) as a function of its position on the CCD (*a*) and after normalization by use of the values in the overlapping region (*b*). The solid line in (*b*) shows the average intensity values derived by binning of the data and the vertical lines mark the nominal measurement range of the autocollimator.

Fig. 2 ▸ shows the intensity of the reticle image of an autocollimator at the position of each individual slit (colour coded) as a function of its position on the CCD. The panels show the absolute intensity values (*a*) and after normalization using the values in the overlap region (*b*). Note that this autocollimator is a special version for synchrotron metrology applications, using a reticle composed of ten slits instead of four as in the standard version of the autocollimator. The solid line in (*b*) shows the average intensity values obtained by binning the data which is then used to derive the flat-field correction. The vertical lines in (*b*) mark the nominal measuring range of the autocollimator of ±1000 arcsec.

**Figure 3:**
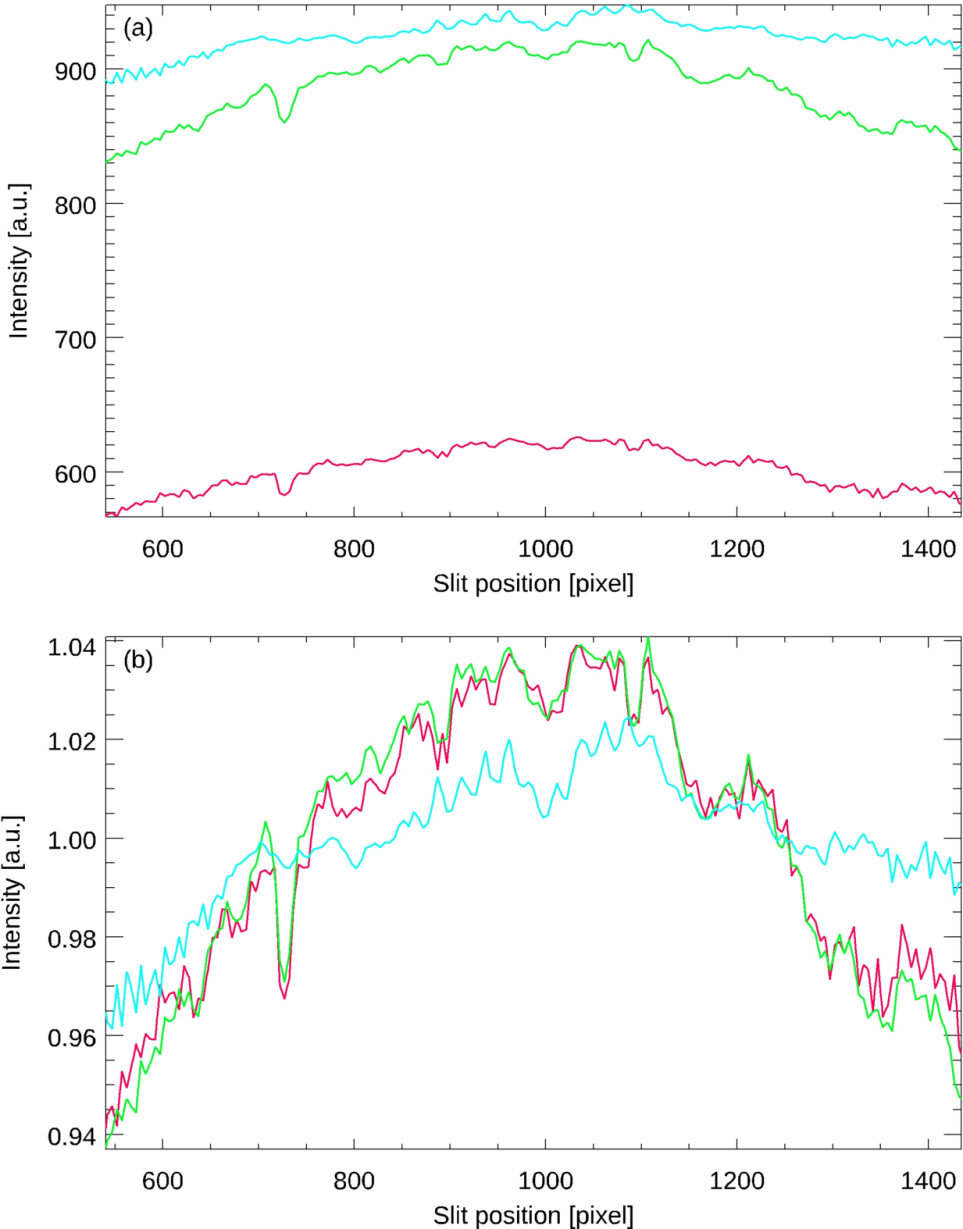
Intensity of the reticle image of the *X*-axis of the autocollimator as a function of its position on the CCD (*a*) and after normalization of the intensities with respect to their average value (*b*). The curves were obtained by use of aperture diameters of 32 mm, 2.5 mm and 1.5 mm (top to bottom curves).

**Figure 4:**
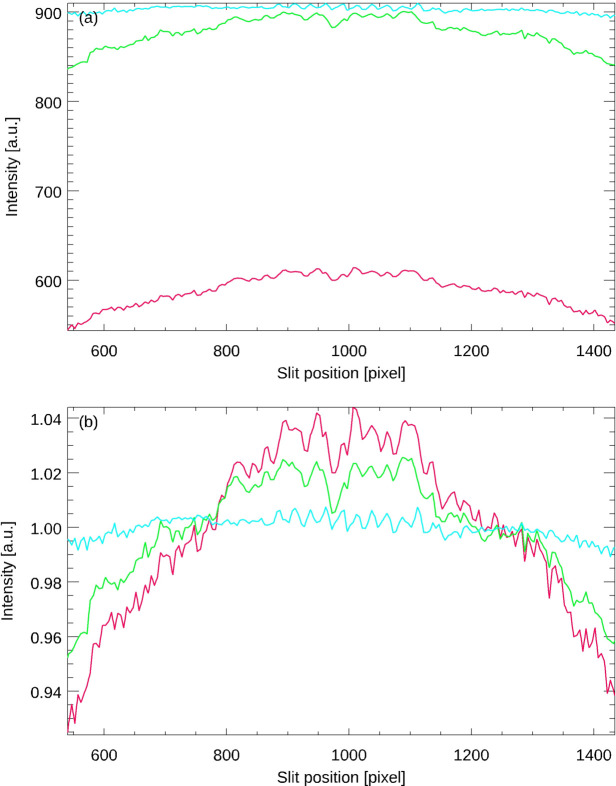
Intensity of the reticle image of the *Y*-axis of the autocollimator from Fig. 3 ▸ as a function of its position on the CCD (*a*) and after normalization of the intensity (*b*).

Fig. 3 ▸ shows the intensity of the reticle image of an autocollimator as a function of its position on the CCD (Elcomat 3000 SN 975, *X*-axis, various apertures, SUT distance 300 mm, measurement range ±1500 arcsec). The three curves were obtained using aperture diameters of 32 mm, 2.5 mm and 1.5 mm (curves from top to bottom). Panel (*a*) shows the absolute intensities and (*b*) shows the same data after normalization of the intensities with respect to their mean values. As expected, the amplitude of the vignetting increases as the aperture size decreases. Note that reproducible changes in image intensity on small spatial scales are also clearly visible. These can be attributed to differences in quantum efficiency between the CCD pixels and minute dust particles in the imaging beam path within the autocollimator, *e.g.* dust particles on the protective glass that is usually placed in front of CCD or CMOS chips. It is expected that these effects will become less pronounced at larger aperture sizes as the beam covers larger volumes of the autocollimator optics, resulting in an averaging effect. Fig. 4 ▸ shows the corresponding data obtained for the *Y*-axis of the same autocollimator under the same general measuring conditions.

### 3.3. Imaging distortion correction

In optical imaging, the optical aberrations of the lens cause geometric distortions of the image, which can be measured and corrected. As the graphical representation of the geometric distortions themselves is of less interest, we limit ourselves to directly demonstrating the effect of the distortion correction on the autocollimator calibration curve in Section 4. In this section, we restrict ourselves to showing the results for the data set with the largest distortion correction as an exemplary case.

**Figure 5:**
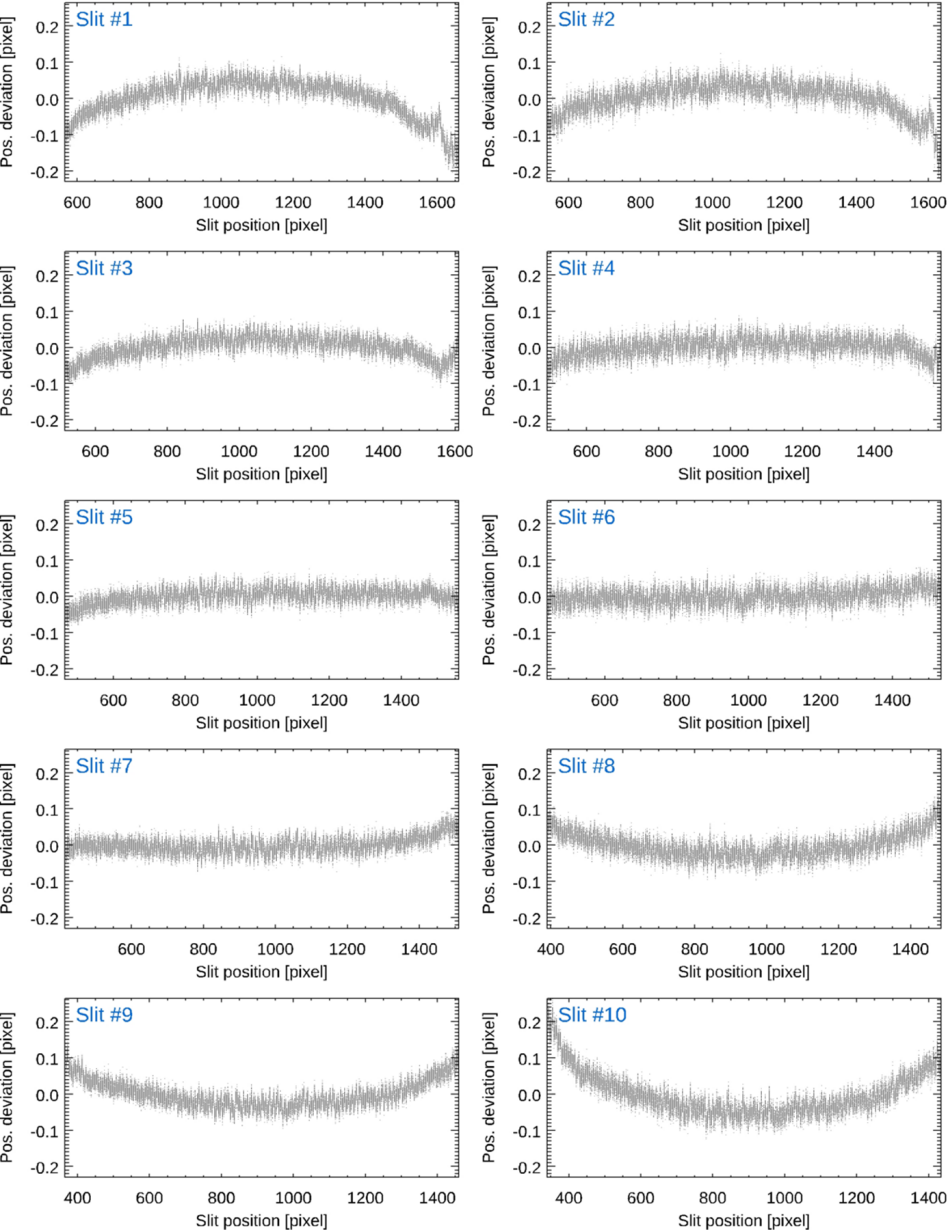
Deviation of the position of each individual slit of the reticle image of an autocollimator from the average position as a function of its position on the CCD. The aperture diameter was 32 mm and the measurement range was ±1500 arcsec.

**Figure 6:**
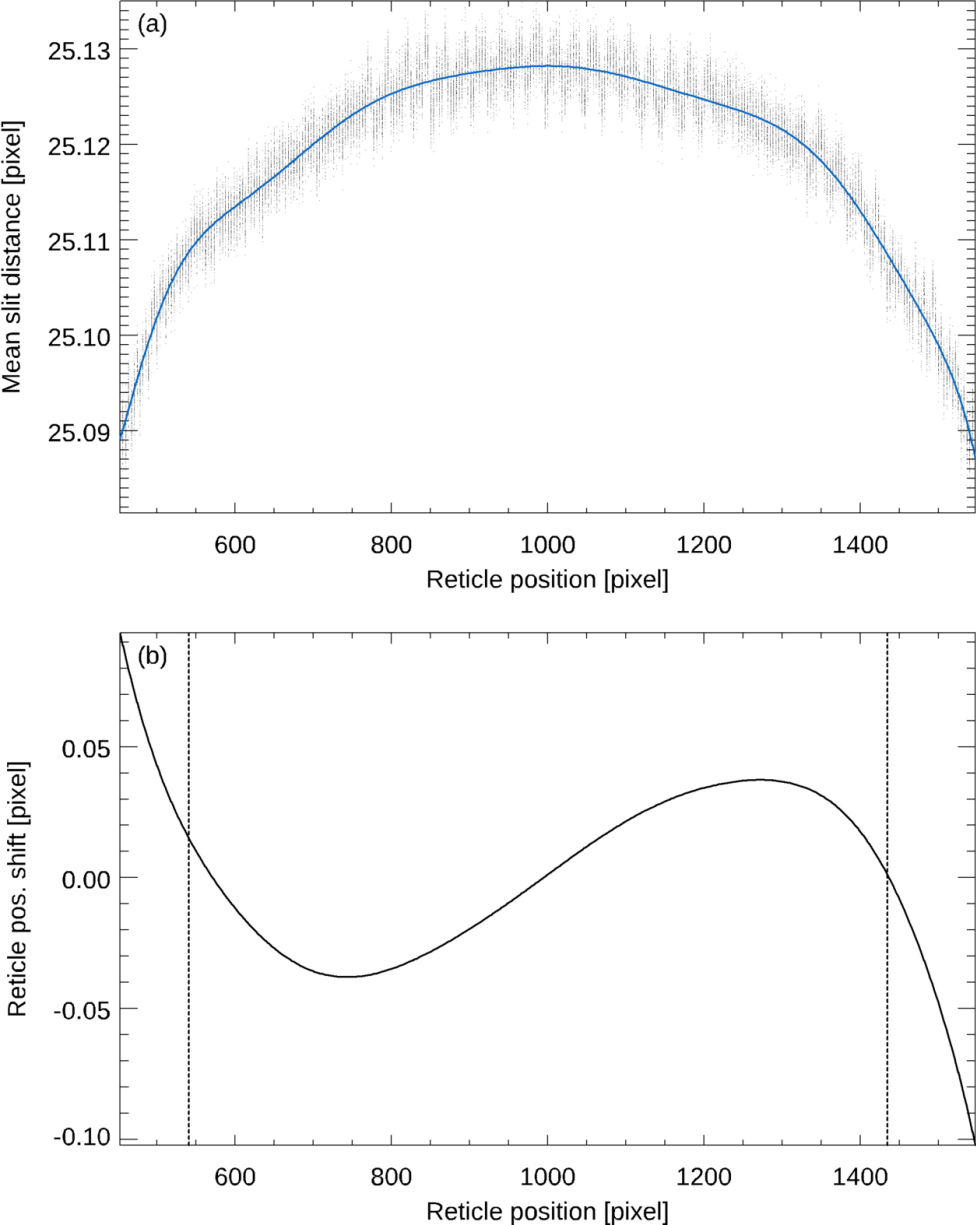
Average distance of the slits of the reticle as a function of its position on the CCD (*a*), derived from the data presented in Fig. 5 ▸, and the distortion correction calculated from the data by integration (*b*). The vertical lines in (*b*) mark the nominal measuring range of the autocollimator of ±1000 arcsec.

Section 3.1 describes in detail how we determine the position of each slit of the reticle image. Fig. 5 ▸ shows the deviation of the position of each individual slit of the reticle image of an autocollimator from the average position as a function of its position on the CCD (Elcomat 3000 SN 975, *X*-axis, aperture 32 mm, SUT distance 300 mm, measurement range ±1500 arcsec). The measurement range was extended to ±1500 arcsec again in order to maximize the effects to be studied. For each reticle position, the average distance between the reticle slits was determined by fitting a linear function to the positions of the slits. The average slit distance was assumed to be proportional to the imaging scale at the corresponding reticle position. A polynomial of order 12 was fitted to the data and integrated to derive the difference between the reticle position with and without imaging distortion. This correction was then applied to the measured reticle position. Fig. 6 ▸ shows the variation of the average distance of the slits of the reticle as a function of its position on the CCD (*a*) which was derived from the data presented in Fig. 5 ▸ and the distortion correction (*b*) calculated from the data. The vertical lines in (*b*) mark the nominal measuring range of the autocollimator of ±1000 arcsec.

## 4. Experimental results

In this section we compare the angle measurements provided by the algorithms built into the autocollimators by the manufacturer with the angle measurements obtained by applying our algorithms. To this end, the autocollimator measurements are compared with those obtained from a reference standard at a national metrology institute in the course of a traceable calibration. At PTB, autocollimator calibrations are performed at the lowest uncertainty level using its primary angle standard, the Heidenhain WMT 220 angle comparator (Probst *et al.*, 1998 ▸). Using various self- and cross-calibration techniques, the standard measurement uncertainty (68% coverage probability, see https://www.bipm.org/documents/20126/2071204/JCGM_100_2008_E.pdf) of the WMT 220 was reduced to *u* = 0.001 arcsec (5 nrad) (Just *et al.*, 2009 ▸; Geckeler *et al.*, 2006 ▸, 2014 ▸). The standard uncertainty of the angle measuring deviations of the autocollimator is usually dominated by the repeatability of the angle measurement of the autocollimator itself. For highly stable autocollimators, calibrations with standard uncertainties down to *u* = 0.003 arcsec (15 nrad) have been achieved (Just *et al.*, 2003 ▸; Geckeler *et al.*, 2010 ▸; Geckeler & Just, 2008 ▸). However, for the small apertures used in deflectometric profilometry, larger uncertainties are usually observed. Even better calibration results have been achieved by using a shearing approach, which is able to separate the measurement errors of the autocollimator and of the reference standard (Geckeler & Just, 2014 ▸).

### 4.1. Data set #1 (Elcomat 3000 SN 975, *X*-axis, aperture 3 mm, SUT distance 300 mm, measurement range ±1500 arcsec)

With the Elcomat 3000 series of autocollimators, the use of the full aperture corresponds to an illuminated aperture of the outgoing measuring beam of approximately 32 mm in diameter, while the diameter of the autocollimator’s objective is 50 mm. These measurement conditions are representative of applications for most users in mechanical engineering and optics and are covered by the manufacturer’s specification of the device. The angular range was restricted to ±1000 arcsec as specified by the manufacturer for the Elcomat 3000 autocollimator.

**Figure 7:**
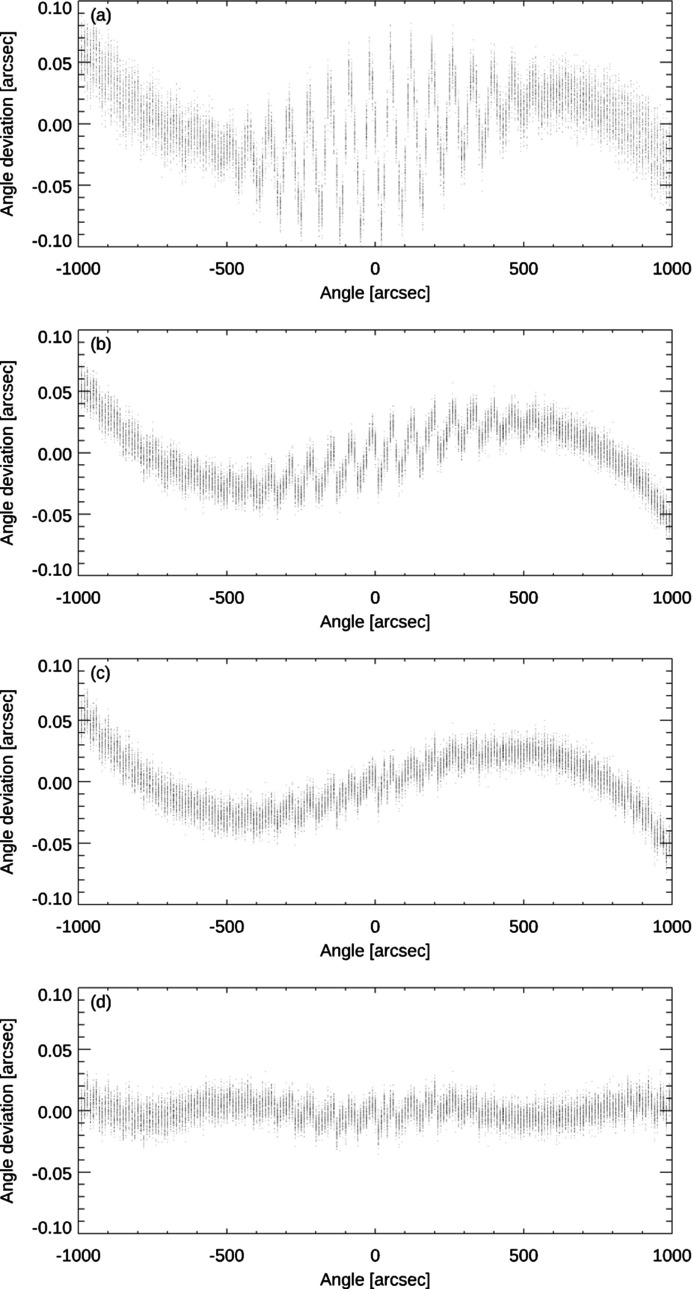
Angle measuring deviations of the autocollimator as a function of the measured angle at full aperture. (*a*) Application of the internal algorithms provided by the manufacturer. (*b*) Application of our CIRIL algorithm. (*c*) CIRIL algorithm and additional flat-field correction. (*d*) CIRIL algorithm and flat-field and imaging distortion corrections.

Fig. 7 ▸ shows the angle measuring deviations of the autocollimator (*i.e.* the difference between the angle measurement of the autocollimator and the reference values provided by our primary angle standard WMT 220) as a function of the measured angle. Plot (*a*) shows the angle measuring deviations of the autocollimator obtained by use of its internal algorithms provided by the manufacturer while (*b*) shows the deviations obtained by use of our CIRIL algorithm from Section 2.1 with *N* = 10 subpixels. Further plots demonstrate the additional improvements achievable by the application of our RISC self-calibration procedures, the flat-field correction from Section 3.2 and the imaging distortion correction from Section 3.3. Plot (*c*) shows the angle measuring deviations of the autocollimator by use of the CIRIL algorithm after the application of the flat-field correction. For this purpose, the intensity values of the reticle images were corrected as described in Section 3.2 and the image analysis by use of our CIRIL algorithm was repeated. Plot (*d*) demonstrates the deviations when both the flat-field correction and an additional imaging distortion correction are applied. In this case, the correction was not applied to the reticle images themselves, but the angular measurement values from plot (*c*) were corrected to take into account the geometric distortion as described in Section 3.3.

The application of our CIRIL algorithm for the accurate localization of the reticle image on the CCD detector described in Section 2 already reduces the measuring deviations of the autocollimator substantially, see plot (*b*) as compared with (*a*). This is especially the case for the quasi-periodic deviations of the autocollimator which are most prominent at the centre of the measurement range and which are characteristic for this type of autocollimator when it is used at full aperture. The first of our additional RISC self-calibration procedures, the flat-fielding described in Section 3.2, did not reduce the amplitude of the measuring deviation on large angular scales. It resulted, however, in markedly smoother deviation curves on medium angular scales, see plot (*c*). The second approach, the imaging distortion correction described in Section 3.3, resulted in a substantial decrease in the angle deviation on large angular scales, see plot (*d*). Therefore, the two RISC approaches complement each other in reducing angle measuring deviations of autocollimators for a broad range of angular scales.

### 4.2. Data set #2 (Elcomat 3000 SN 975, *X*-axis, aperture 2.5 mm, SUT distance 300 mm, measurement range ±1500 arcsec)

This data set was obtained using a circular aperture with a diameter of 2.5 mm. It represents the use case of deflectometric profilometry for accurate form measurement of beam shaping optics of synchrotrons and X-ray free-electron lasers. Here, the beam footprint on the SUT is limited by an aperture to increase the lateral resolution (Lacey *et al.*, 2019 ▸). Note that this aperture is much smaller than the aperture sizes specified for the autocollimator by the manufacturer.

**Figure 8:**
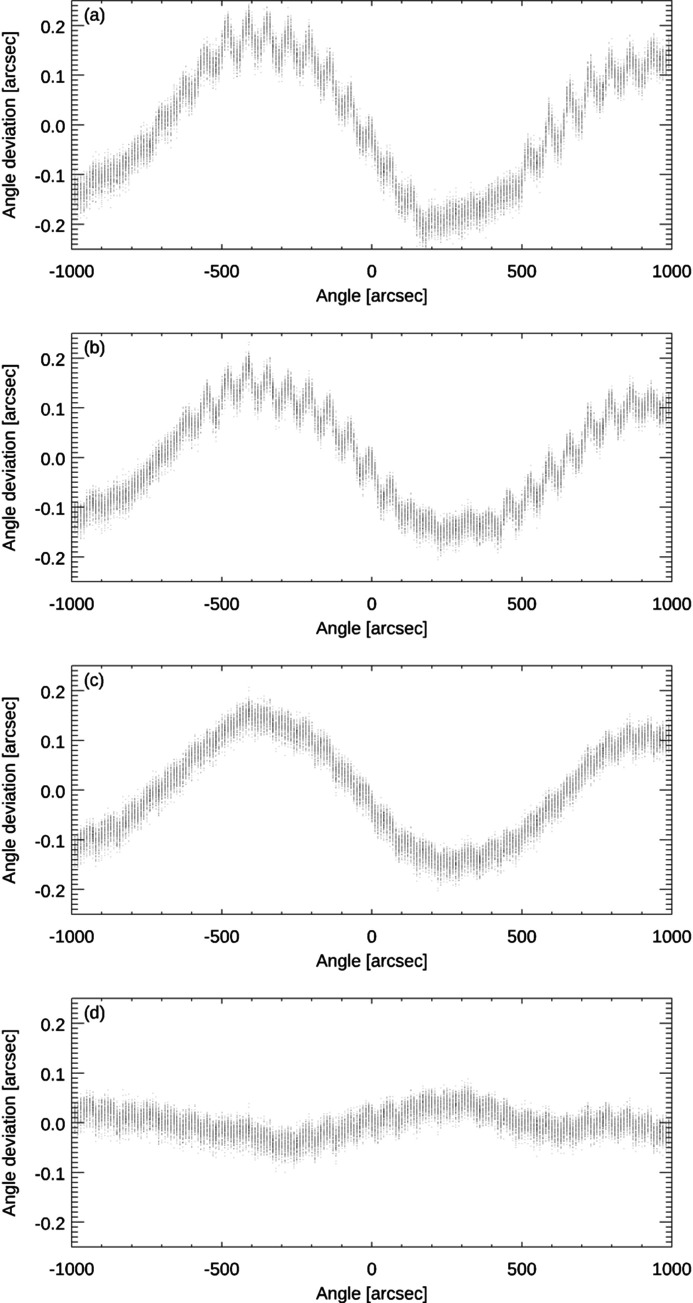
Angle measuring deviations of the autocollimator as a function of the measured angle at an aperture of 2.5 mm. (*a*) Application of the internal algorithms provided by the manufacturer. (*b*) Application of our CIRIL algorithm. (*c*) CIRIL algorithm and additional flat-field correction. (*d*) CIRIL algorithm and flat-field and imaging distortion corrections.

Fig. 8 ▸ shows the corresponding data of the autocollimator at this aperture, whereby the figure legend corresponds to that of Fig. 7 ▸.

Finally, we summarize the results of the application of our algorithms to the autocollimator calibration data presented in this section, as well as additional data sets. Table 1 ▸ summarizes the error reduction achieved by applying our CIRIL reticle image localization algorithm and our RISC flat-fielding and geometric image distortion corrections. The standard deviation of the angle measurement deviation of the autocollimator with respect to the reference values provided by our primary angle standard WMT 220 is calculated. The error ratio is defined as the standard deviation which results from the application of our algorithms divided by the standard deviation results from the application of the autocollimator’s internal algorithms. As expected, the smallest error ratio of 0.21–0.43 (*i.e.* the largest error reduction by the application of our algorithms) is seen for the largest angular measurement range of ±1500 arcsec. For the smaller measurement ranges of ±30 arcsec and ±4 arcsec, the ratio is still 0.50–0.88 and 0.66–0.88, respectively.

Note that the measurement range of the autocollimator specified by the manufacturer is ±1000 arcsec and that we have increased the range beyond this in order to maximize the effects to be studied. Furthermore, the autocollimator is also used with small apertures that are considerably smaller than those specified by the manufacturer.

We would like to emphasize again that this approach is based on the analysis of a set of reticle images without recourse to external measurements. In the case where external reference measurements are used, the application of our approach is nevertheless advantageous. On the one hand, the influence of random errors is reduced, so that fewer measurements need to be averaged to achieve the same signal-to-noise ratio. On the other hand, the systematic errors on different angular scales are reduced, so that the interpolation of the calibration curves between their sampling points is less error prone.

## 5. Conclusions

We have developed an original algorithm for determining the position of a reticle image on a CCD detector that is capable of significantly reducing systematic errors in position measurement. In addition, we have developed self-calibration procedures to correct for errors such as intensity changes and geometric distortions that are part of the imaging process. We demonstrated that these corrections can be derived from a redundant set of images without recourse to external calibration data. As a proof-of-concept test, we applied the new algorithms to reticle image data from a commercial autocollimator taken during autocollimator calibrations performed at PTB with its primary angle standard WMT 220. Using 18 calibrations over different angular ranges (±1500 arcsec, ±30 arcsec and ±4 arcsec) and with different apertures (32 mm, 2.5 mm and 1.5 mm) to limit the beam diameter of the autocollimator, we have shown that our algorithm can produce results that are more accurate than those of the autocollimator’s built-in algorithm. The standard deviation of the autocollimator’s angle measuring deviations from the reference values provided by the primary angle standard WMT 220 was used to assess the capabilities of our new algorithms. For the 2.5 mm-diameter aperture, which is the most commonly used in autocollimator-based deflectometric profilometry, the standard deviation of the angle measurement errors could be reduced by a factor of four to five (compared with the autocollimator’s built-in algorithms) over its nominal measurement range of ±1000 arcsec. We believe that the results are crucial for reaching fundamental metrological limits in deflectometric profilometry using state-of-the-art electronic autocollimators. Although we have used image data from an autocollimator to demonstrate their practical capabilities, our algorithms are applicable to a wide range of cases where a pattern is imaged by an objective onto a detector with limited pixel resolution. It is therefore not restricted to a particular reticle design or to a particular type of instrument, such as autocollimators. We therefore see potential for improving metrology in a wide range of related applications.

## Figures and Tables

**Table 1:** Error reduction achieved by our CIRIL algorithm and RISC approaches

Aperture (mm)	Measurement range (arcsec)	Sampling step (arcsec)	Error ratio	Axis	Remarks
32	3000†	10	0.27	*X*	Sampling step > pixel-scale (*ca*. 2.4 arcsec)‡
32	60	0.2	0.50	*X*	
32	8	0.2	0.67	*X*	
5	60	0.2	0.74	*X*	
2.5	3000†	10	0.21	*X*	Sampling step > pixel-scale
2.5	60	0.2	0.81	*X*	
2.5	8	0.2	0.82	*X*	
1.5	3000†	10	0.41	*X*	Sampling step > pixel-scale
1.5	60	0.2	0.85	*X*	
1.5	8	0.2	0.79	*X*	
32	8	0.2	0.74	*Y*	
2.5	3000†	10	0.20	*Y*	Sampling step > pixel-scale
2.5	8	0.2	0.82	*Y*	
1.5	3000†	10	0.43	*Y*	Sampling step > pixel-scale
1.5	60	0.2	0.88	*Y*	
1.5	8	0.2	0.73	*Y*	

Summary					
1.5–32	3000†	10	0.31 (0.21–0.43)	*X* and *Y*	CIRIL algorithm + flat-fielding + imaging distortion correction
1.5–32	60	0.2	0.76 (0.50–0.88)	*X* and *Y*	CIRIL algorithm + imaging distortion correction§
1.5–32	8	0.2	0.76 (0.66–0.88)	*X* and *Y*	CIRIL algorithm + imaging distortion correction§

† Restricted to the nominal measurement range of ±1000 arcsec for the analysis.

‡ In these cases, the sampling step is larger than the pixel scale of the CCD.

§ In the case of the smaller measurement ranges, the reticle images did not overlap sufficiently for the flat-field correction to be calculated. The correction for the wider measurement range could have been used, but this would have violated the principle of deriving the correction from each data set independently.
